# Complex of human Melanotransferrin and SC57.32 Fab fragment reveals novel interdomain arrangement with ferric N-lobe and open C-lobe

**DOI:** 10.1038/s41598-020-79090-8

**Published:** 2021-01-12

**Authors:** Kristyn Hayashi, Kenton L. Longenecker, Yi-Liang Liu, Bryan Faust, Aditi Prashar, Johannes Hampl, Vincent Stoll, Sandro Vivona

**Affiliations:** 1 Research and Development, AbbVie Inc., South San Francisco, CA 94080 USA; 2 Research and Development, AbbVie Inc., North Chicago, IL 60064 USA

**Keywords:** Biochemistry, Biological techniques, Biophysics, Biotechnology, Cancer, Cell biology, Chemical biology, Drug discovery, Molecular biology, Physiology, Structural biology, Diseases, Medical research, Molecular medicine, Oncology

## Abstract

Melanotransferrin (MTf) is an iron-binding member of the transferrin superfamily that can be membrane-anchored or secreted in serum. On cells, it can mediate transferrin-independent iron uptake and promote proliferation. In serum, it is a transcytotic iron transporter across the blood–brain barrier. MTf has been exploited as a drug delivery carrier to the brain and as an antibody-drug conjugate (ADC) target due to its oncogenic role in melanoma and its elevated expression in triple-negative breast cancer (TNBC). For treatment of TNBC, an MTf-targeting ADC completed a phase I clinical trial (NCT03316794). The structure of its murine, unconjugated Fab fragment (SC57.32) is revealed here in complex with MTf. The MTf N-lobe is in an active and iron-bound, closed conformation while the C-lobe is in an open conformation incompatible with iron binding. This combination of active and inactive domains displays a novel inter-domain arrangement in which the C2 subdomain angles away from the N-lobe. The C2 subdomain also contains the SC57.32 glyco-epitope, which comprises ten protein residues and two *N*-acetylglucosamines. Our report reveals novel features of MTf and provides a point of reference for MTf-targeting, structure-guided drug design.

## Introduction

Melanotransferrin (MTf) is a 75 kDa member of the transferrin superfamily, which comprises single chain, iron-binding glycoproteins responsible for iron homeostasis in cells^1–3^. Within the transferrin superfamily, there are numerous reported structures of both the iron-bound and apo forms of serum transferrin, lactoferrin and ovotransferrin^4,5^. Transferrins generally contain two paralog domains called the N- and C-lobes, each comprising two subdomains (N1–N2 and C1–C2) of roughly 170 amino acids connected by two beta-strands. Each lobe is capable of binding iron extracellularly and of releasing it in a pH-dependent manner along the endocytic pathway^6–11^. In the transferrin domain, iron binds as a bidentate carbonate complex and is coordinated by four protein ligands at the cleft of the N1–N2 and C1–C2 subdomains: an Aspartate and a Histidine in subdomain 1 and two Tyrosines in subdomain 2^12^. The bidentate carbonate is in turn coordinated by an Arginine and a Threonine in subdomain 2. Upon iron binding, transferrin domains undergo a conformational change, rotating from an open to a closed state, enabling the Aspartate and Histidine to contact the iron atom^13–17^. Unlike most transferrins, MTf binds only one iron atom through its N-lobe^18,19^ with an apparent affinity of 4.4 × 10^17^ M^−1^^20^ and has also been predicted to have a zinc-binding site^21^. In addition, MTf is expressed as either a secreted or a glycosylphosphatidylinositol (GPI)-anchored form via alternative mRNA splicing^22,23^.

MTf was first discovered as the tumor-associated antigen (p97) in melanoma where it promotes tumor proliferation, migration, angiogenesis and differentiation^24–36^. In normal tissues, MTf (also known as CD228 and MFI2) is primarily expressed in the salivary glands, skin, kidney and ducts of sweat glands^37^. Modulation of its expression affects genes involved in membrane transport, energy metabolism, cell proliferation and survival^38^. Due to its iron-binding properties and homology to serum transferrin, MTf was initially explored as an additional iron transporter with Kennard et al. demonstrating iron uptake in CHO cells by membrane-anchored MTf^39–41^. In addition, MTf in serum appears to be actively transcytosed across the brain epithelium 10–15 times faster than lactoferrin or serum transferrin^42^, thus providing iron to the central nervous system (CNS)^43^. MTf has been used to deliver several drugs to the brain. For example, it has been conjugated to the chemotherapeutic agents Paclitaxel and Adriamycin^44^ to intracranially target glioma and mammary tumors, produced as a chimera with the antibody Trastuzumab^45^ to target breast cancer metastasis in the brain, fused to the coxsackie-adenovirus receptor to perform adenovirus-based gene delivery^46^, and used as a short peptide (i.e. DSSHAFTLDELR) conjugated to interleukin 1 receptor antagonist (IL-1RA) to treat neuropathic pain^47^. In brain, elevated levels of secreted MTf have been associated with Alzheimer’s disease, as iron overload has been shown to accelerate ß-amyloid production^48–54^. However, MTf does not appear essential for iron homeostasis and may have other functions^55–61^ such as plasminogen activation in cell migration^62,63^.

Besides in melanoma, MTf expression was found elevated in colorectal cancer^64^ and triple-negative breast cancer (TNBC) patient-derived xenografts and primary tumor specimens^65^ (EP: 1,120,651). Anti-MTf antibody-drug conjugates (ADCs) delivering DNA-damaging pyrrolobenzodiazepines (PBDs) demonstrated tumor regression in TNBC patient-derived xenograft models^65^. One of these ADCs, SC-005, completed dose escalation in a phase I clinical trial, where SC57.32, the native, murine version of SC-005, was used as an immunohistochemistry tool to identify MTf in patient samples (NCT03316794).

Here we report the novel crystal structure of MTf bound to the SC57.32 Fab fragment, providing insights into iron binding, revealing a novel N- and C-lobe interaction and, in combination with Surface Plasmon Resonance, elucidating the binding mechanism and kinetics of SC-005/SC57.32 to a glyco-epitope. This study provides a structural reference for future MTf-targeting applications.

## Materials and methods

### Protein expression and purification

The sequence of the cynomolgus monkey MTf protein was deduced by running a Basic Local Alignment Search Tool (BLAST)^66^ on the DNA sequence encoding the human MTf open reading frame (derived from NCBI: NM_005929) on an exon by exon basis versus the cynomolgus whole genome shotgun contigs database at the National Center for Biotechnology Information. A monkey open reading frame was manually assembled, assuming conservation of exon boundaries between species, in light of known splice donor/acceptor sequence rules. The sequence of the individual cynomolgus exons was independently confirmed by PCR amplification of the exons from cynomolgus genomic DNA and Sanger sequencing of the products.

cDNA for expression of recombinant human MTf(20-709) (NCBI: NP_005920), cynomolgus monkey MTf(20-709) and rat MTf(20-709) (NCBI: NM_001105872) extracellular domains was constructed by synthesis of the three respective amplicons followed by subcloning into a CMV driven expression vector, based on the pEE12.4 vector, downstream of an IgK signal peptide leader sequence. Proteins were produced as secreted polyhistidine-tagged fusion proteins by transient transfection of suspension CHO-S cells (ThermoFisher Scientific) using the Maxcyte STX electroporation system. Cells were resuspended in Maxcyte electroporation buffer at 2E8 cells/mL, electroporated using the CHO cell type setting, allowed to recover without shaking for 40 min at 37 °C and then resuspended in CD OptiCHO media (ThermoFisher Scientific) at 4E6 cells/mL. For crystallization studies, human MTf cultures were supplemented with 10 mM mannosidase I inhibitor kifunensine (Sigma-Aldrich) at the time of transfection to favor formation of high mannose N-glycans^67^. The proteins were first purified from cell culture supernatant via immobilized metal affinity chromatography and then eluted over a Superdex-200 size exclusion chromatography (SEC) column (Cytiva Life Sciences) equilibrated with 10 mM HEPES buffered saline, pH 7.4 (HBS) and equipped with a multiangle light scattering (MALS) setup (Optilab T-rEX and Treos, Wyatt). Fractions of the monomeric, monodisperse peak were combined, and the concentration determined via UV-spectroscopy and differential refractometry. No iron was supplemented during protein preparation. Lyophilized polyhistidine-tagged mouse MTf(20-708) (NCBI: NP_038928) protein was purchased from Sino Biological and reconstituted at 0.5 mg/mL in PBS, pH 7.4.

Anti-MTf murine antibody SC57.32^65^ was produced from hybridoma cells and purified using Protein A MabSelect SuRe (Cytiva Life Sciences), followed by papain digestion using a Fab preparation kit (Thermo Fisher Scientific) and concluded by SEC-MALS polishing as described above.

### Surface plasmon resonance

Purified human and cynomolgus monkey MTf proteins were diluted in HBS supplemented with 0.05% (v/v) Polysorbate 20 (PS20) and 3 mM EDTA (HBS-EP+ buffer) to concentrations of 2.5, 5, 10, 20, 40 and 80 nM. Rat and mouse MTf proteins were diluted in HBS-EP+ at 1000 nM concentration to rule out lower affinity binding. Data were collected on a Biacore T200 (Cytiva Life Sciences) equipped with a CM5 chip conjugated with anti-mouse Fc capture antibodies, as directed. SC57.32 murine antibody was captured at a low surface density (34 RU average across cycles), followed by injection of monomeric MTf protein dilutions at a flow rate of 40 µL/min for 90 s of association and 360 s of dissociation. After each injection, the chip surface was then regenerated with 10 mM glycine–HCl, pH 1.5 (180 s at 50 µL/min). Data were processed with Biacore T200 Evaluation Software and globally fit with a 1:1 Langmuir binding model (bulk shift set to zero) to extract kinetics and affinity constants (k_a_, k_d_, K_D_).

### Protein X-ray crystallography

A 1:1 complex of human MTf with SC57.32 Fab was purified via SEC-MALS after incubation of a MTf:Fab 1:2 molar ratio mixture (Supplementary Fig. S1). Complex of MTf with SC57.32 Fab was concentrated to 10 mg/mL using a 50 kDa Amicon (Millipore EMD). The final co-crystallization was carried out by mixing 100 nL of the concentrated human MTf/SC57.32 Fab complex with 100 nL of precipitant (100 mM sodium acetate, pH 4.6 and 30% PEG300) on a Mosquito instrument (TTP Labtech) at room temperature. Crystals appeared within a week and grew to full size within two weeks (Supplementary Fig. S1). After adding 30% ethylene glycol for cryo protection, 0.1 mm and 0.2 mm crystal loops (Hampton Research) were used for harvest and crystals were flash frozen in liquid nitrogen. No iron was superficially supplemented during protein preparation or crystallization. Crystals diffracted to d_min_ = 3.06 Å. X-ray diffraction data were collected at the APS IMCA 17-ID beamline using a Pilatus 6 M detector. The data were processed using XDS (Kabsch) and autoPROC^68^ and Bijvoet pairs were retained for anomalous difference map calculations. The MTf structure was solved by molecular replacement using Phaser^69^ with coordinates for diferric mare lactoferrin from PDB entry 1B1X^70^ as the search model. The structures were refined against the diffraction data using BUSTER^71^ software from Global Phasing Ltd and iterative graphical refinement using COOT^72^. The coordinates for MTf/SC57.32 Fab complex have been submitted with PDB code 6XR0. Structures were analysed and figures were prepared using MOE (Chemical Computing Group) and PyMol (Schrodinger, LLC).

## Results

**Figure 1:**
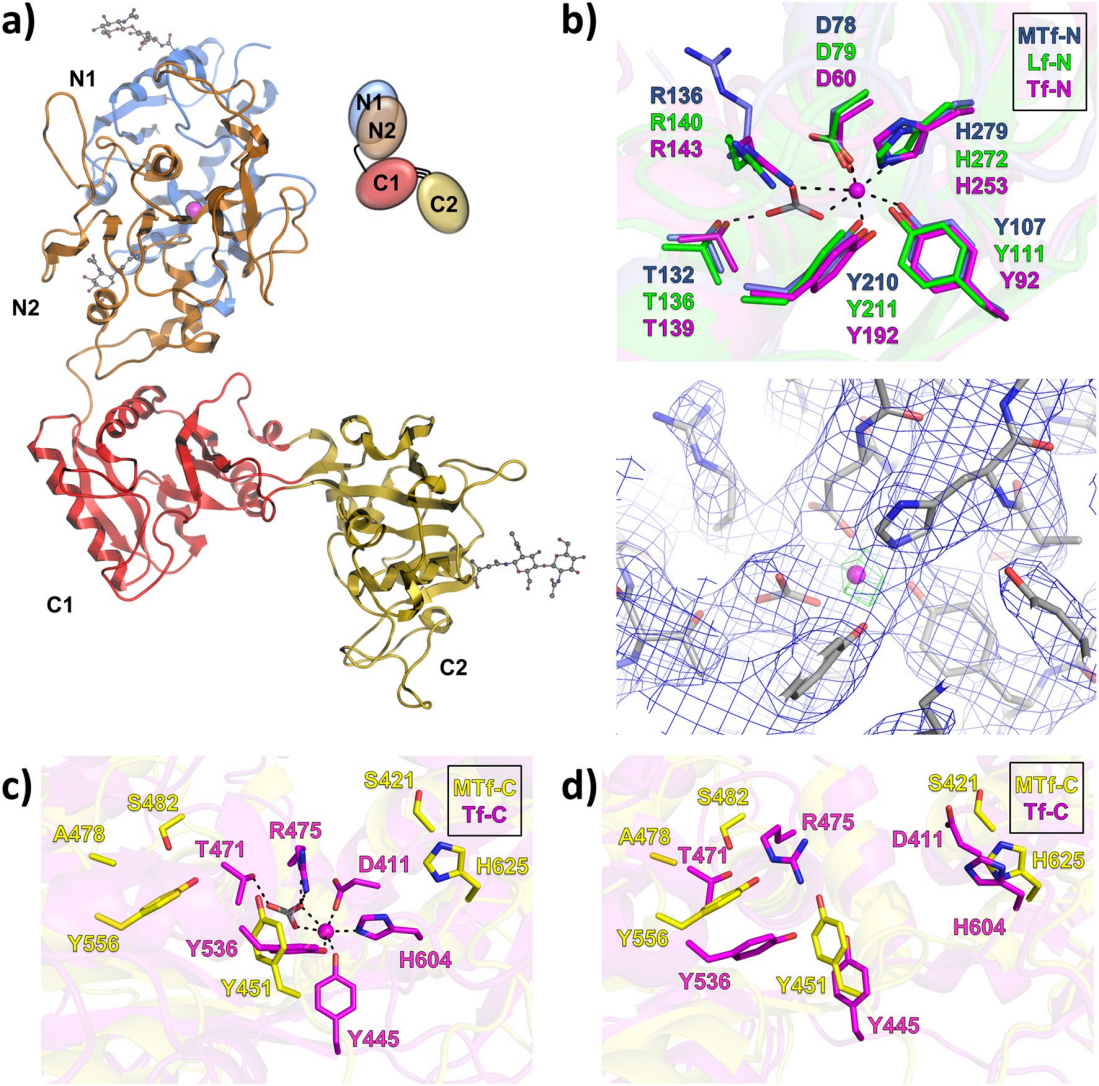
Transferrin fold and iron coordination. (**a**) Structure of human melanotransferrin (MTf, PDB: 6XR0) shows a transferrin fold featuring an iron-bound (magenta sphere) N-terminal lobe with N1 (blue) and N2 (orange) subdomains in closed conformation and an iron-free C-terminal lobe with C1 (red) and C2 (yellow) subdomains in an open state. MTf contains three N-glycans (gray sticks) at N38, N135 and N515. (**b**) Top: MTf N-lobe (blue sticks) coordinates iron (magenta sphere) and carbonate (gray sticks) via connecting coordination bonds (black dashes). Corresponding lactoferrin (green sticks, PDB: 1LFG) and serum transferrin (magenta sticks, PDB: 3V83) residues are overlaid. Bottom: MTf (gray sticks) with carbonate (gray sticks) and iron (magenta sphere) bound. The 2Fo-Fc map is contoured at 1σ (blue mesh) and an anomalous difference Fourier map contoured at 8σ (green mesh). (**c**) Iron coordination residues of closed, iron-bound C-lobe of serum transferrin (magenta sticks, PDB: 3V83) and open, inactive MTf C-lobe (yellow sticks). (**d**) Open and iron-free C-lobe overlay between active serum transferrin (magenta sticks, PDB: 2HAV) and inactive MTf (yellow sticks).

Here we report the novel structure of MTf (PDB: 6XR0, Fig. 1A), which was solved by molecular replacement to a resolution of 3.06 Å and refined to an R-factor of 20.2 (R_free_ = 22.5). Crystallographic statistics are summarized in Table 1. Our MTf structure shows unique features compared to other members of the transferrin superfamily and can explain previous reports regarding MTf iron binding properties.

**Table 1:** Data collection and refinement statistics.

X-ray diffraction data	MTf-FabSC57 Complex
PDB ID	6XR0
Space group	P2_1_
Unit cell lengths (a, b, c)	58, 137, 111
Unit cell angles (α, β, γ)	90, 91.5, 90
Resolution (Å)	3.06 (3.06–3.12)
Observations	217,335
Unique	32,506
Completeness (%)	99.8 (99.9)
Mean diffraction signal (I/σ)	12 (2.2)
*R*_pim_ (%)	8.4 (62)
*R*_merge_ (%)	13.2 (99)
Redundancy	6.7 (6.8)
**Model refinement**	
Reflections (work/free)	30,778/1728
*R*_factor_ (work/free %)	20.2/22.5
Protein atoms	8545
Waters	50
Mean B value	71
RMSD ideal bond length (Å)	0.008
RMSD ideal bond angles (°)	0.93
Ramachandran (% favored/allowed/outliers)	93.7/6.1/0.2

Values for high resolution shell in parentheses.

### MTf active N-lobe and structural basis for C-lobe inactivity

MTf is composed of two globular domains, typically referred to as the N- and C-lobes, connected by a seven amino acid linker. Each lobe is further comprised of two subdomains, N1–N2 and C1–C2 (Fig. 1A). The active and iron-bound N-lobe shows a closed conformation with one iron atom bound at the N1–N2 cleft. On the other hand, the C-lobe is inactive and incapable of iron binding, displaying an open conformation that would require a C2 rotation of 72.1° to align with the closed conformation of ferric N-lobe (Supplementary Fig. S2). This open angle is the largest in comparison to other transferrin structures^4^. Since MTf contains three N-glycosylation sites (Asn 38, Asn 135 and Asn 515), recombinant MTf was expressed in the presence of mannosidase I inhibitor kifunensine to reduce the glycan complexity and further aid crystallization^67^. The first *N*-acetylglucosamine (GlcNAc) of an N-glycan is clearly defined at all three expected glycosylation sites in our structure, while the second is visible for two out of three sites (Supplementary Fig. S4).

The N-lobe of MTf is consistent with those of iron-bound lactoferrin (PDB: 1LFG)^73^ and serum transferrin (PDB: 3V83)^74^, both in overall fold (RMSD 1.2 and 1.0 Å, respectively) and in iron coordination (Fig. 1B). The MTf N-lobe contains a canonical iron binding tetrad DYYH (Asp 78, Tyr 107, Tyr 210 and His 279) as well as the carbonate anion. While both carbonate protein ligands (Arg 136 and Thr 132) are conserved in the MTf N-lobe, only the hydroxyl of Thr 132 contacts the carbonate whereas the side chain of MTf Arg 136 is oriented towards Asp 61 in our density map. Alternate partial occupancy could be possible, but determination is beyond the resolution of our data. Multiple conformations of Arginine, including side chain conformers angled away from the carbonate, have been observed in other transferrin structures and have been implicated in iron release^75,76^. It is worth noting that the pH of the crystallization condition (i.e. 4.6) is lower than that believed to cause conformation opening and iron release in the endosome (i.e. 5.6)^9,10^. Additionally, iron was not supplemented superficially before or after crystallization, resulting in incomplete iron occupancy (Supplementary Fig. S3). While iron-bound transferrins have been crystallized in acidic conditions (e.g. rabbit serum transferrin at pH 5.4, PDB: 1JNF)^77^, and while anomalous scattering confirms the presence of iron in our structure (Fig. 1B), it is possible our crystal contains some iron-free MTf, which could be reflected by the conformation of Arg 136 and could be supported by the higher B-factor values observed in the N-lobe compared to the C-lobe (Supplementary Fig. S3). On the other hand, the C-lobe may be more rigid due to the binding of the SC57.32 Fab. Additionally, apo MTf may have remained outside the crystal in an open state.

In contrast to the active and iron-bound N-lobe, the C-lobe shows notable differences with its homologs. Comparison of the open MTf C-lobe to the closed, iron-bound C-lobe of serum transferrin (PDB: 3V83)^74^ highlights the difference in conformation (RMSD 7.9 Å, Fig. 1C) that occurs between open/closed states in the geometry of the iron-coordinating tetrad. Comparison of the MTf C-lobe to the iron-free but active C-lobe of serum transferrin (PDB: 2HAV)^78^ shows a similar open lobe conformation (RMSD 2.5 Å) but key differences in specific iron coordination residues (Fig. 1D). Within the canonical iron binding tetrad DYYH, the typical Aspartate (e.g. serum transferrin Asp 392) is instead Ser 421 in MTf. This Aspartate to Serine mutation has been explored in other transferrin homologs and, while it appears to affect the open/closed conformation switch, it does not abolish iron binding^79–84^. On the other hand, variation of the typical carbonate protein ligands Threonine and Arginine (e.g. serum transferrin Thr 471 and Arg 475) with MTf Ala 478 and Ser 482, respectively, abolishes binding of the carbonate and is most likely responsible for the inability of MTf C-lobe to bind iron. While individual point mutants of serum transferrin Thr 471 and Arg 475 displayed impaired iron binding, concomitant mutations at both sites resulted in loss of iron binding in previous studies^84–86^. Thus, the MTf C-lobe is inactive due to deviation of the Aspartate within the iron-binding tetrad and variation of both carbonate protein ligands Threonine and Arginine.

### N- and C-lobe adopt a novel interdomain arrangement

**Figure 2:**
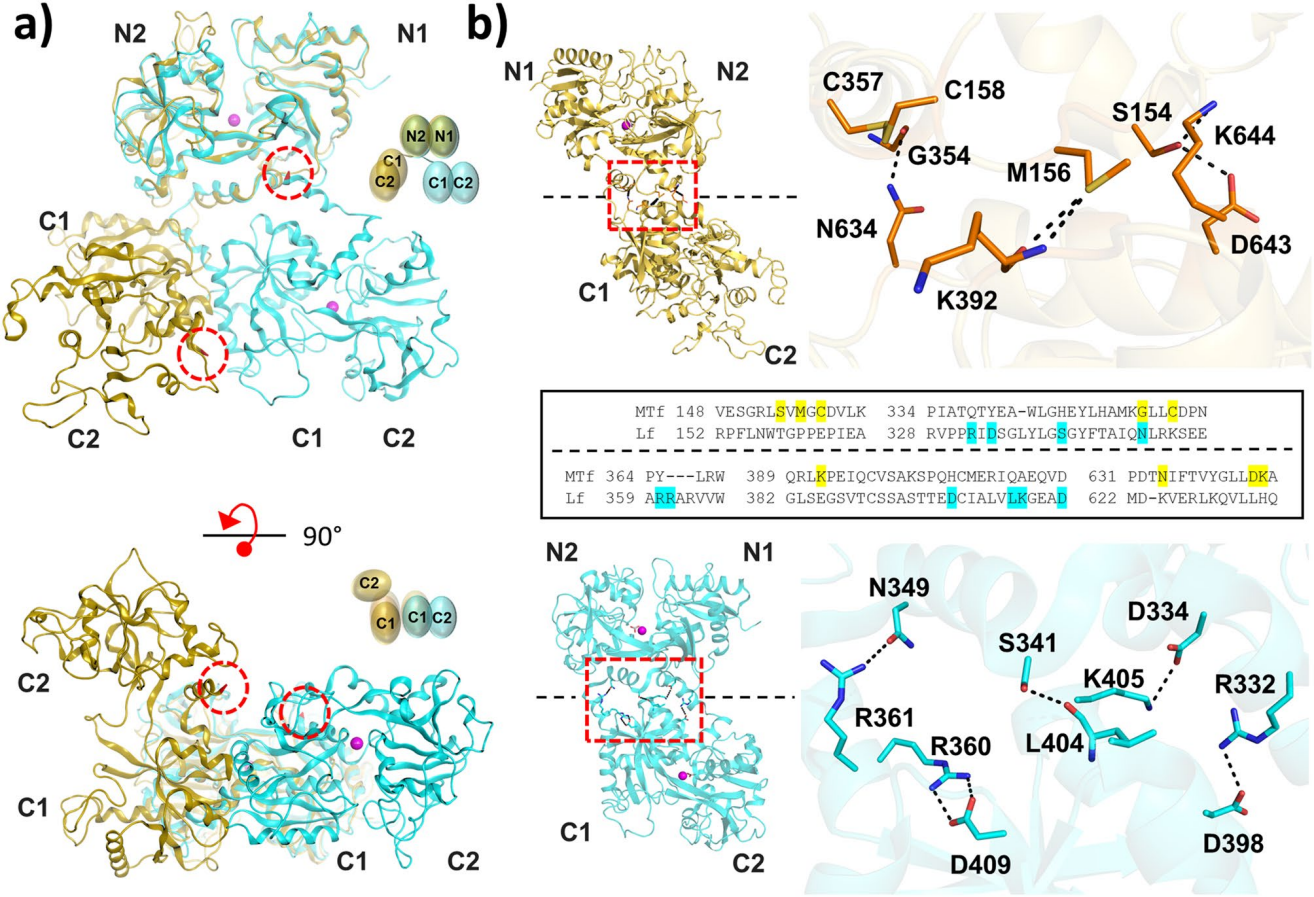
MTf displays divergent N–C-lobe interface (**a**) Top: Iron-bound (magenta spheres) human lactoferrin (cyan cartoon, PDB: 1LFG) aligned with MTf (yellow cartoon) via the N-lobe. C-termini are colored in red and indicated with dashed red circles. Bottom: 90°, x-axis rotation of top panel yielding bottom view of the C-lobes. (**b**) Top and bottom: MTf (orange sticks) and lactoferrin (cyan sticks) N–C interfaces. Hydrogen bonds and salt bridges within 3.8 Å were found using Amoeba force field in MOE (Chemical Computing Group) and are depicted as black dashes. Middle: Amino acid sequence alignment between human MTf and human lactoferrin with highlighted residues connecting the N- and C-lobes within 3.8 Å.

In comparison with other transferrin structures, the MTf structure (PDB: 6XR0) reveals a novel N- and C-lobe arrangement (Fig. 2A). In most transferrins, the C1 subdomain primarily contacts the N1 subdomain and the C-terminus folds back towards the N1 subdomain. While specific N–C contacts are not necessarily conserved across all transferrins, previously published transferrin structures display slightly different solutions to achieve the same global conformation and N–C interface. For example, in human lactoferrin (PDB: 1LFG)^73^ the lobes are oriented by hydrogen bonds between Arg 361 and Arg 360 on the linker to Asn 349 of N2 and Asp 409 of C1, respectively, while additional contacts are made between the N1 and C1 subdomains (Fig. 2B). In contrast, the MTf C1 subdomain contacts the N2 subdomain; for example, Ser 154 forms hydrogen bonds with Asp 643 and Lys 644 (Fig. 2B). The resulting domain arrangement shows the C2 subdomain and its C-terminus distal from the N1 subdomain rather than folding back towards it. As a result, the predicted zinc-binding site by Garrett et al.^21^ is not realized with Glu 413 greater than 15 Å away from the proposed thermolysin-like zinc-binding site. However, multiple MTf N–C states may be possible with MTf adopting a global conformation similar to other transferrins or flexibly sampling different conformations.

### SC-005/SC57.32 targets a glyco-epitope in the C2 subdomain

**Figure 3:**
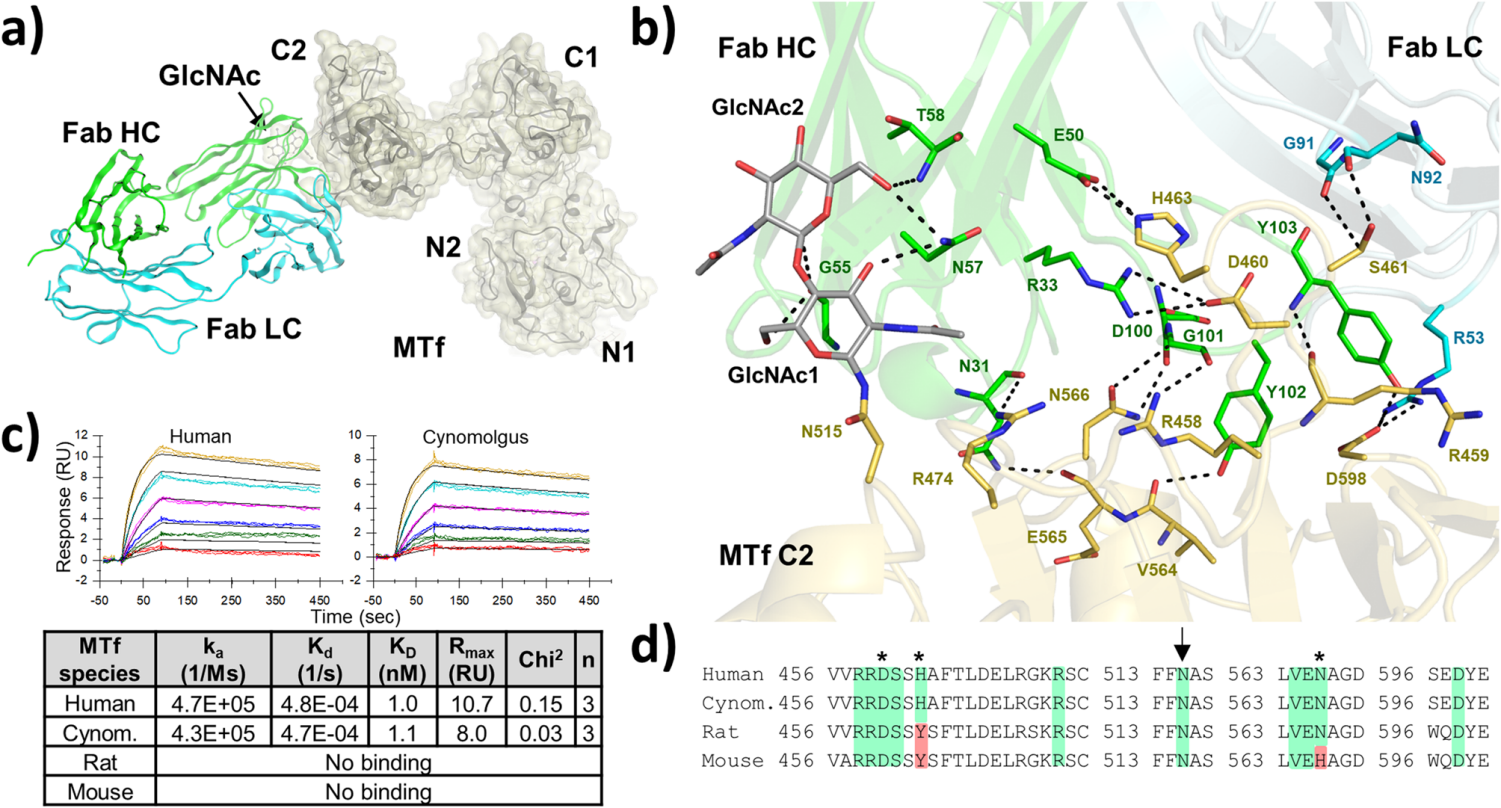
SC57.32/MTf complex and SC-005 species cross-reactivity (**a**) Cartoon representation of MTf (grey cartoon) with yellow surface representation in complex with the SC57.32 Fab fragment (heavy chain in green, light chain in cyan). (**b**) Ten residues (yellow sticks) and one associated N-glycan (light grey sticks) of the MTf C2 subdomain make direct contact with thirteen Fab residues, ten of which are in the heavy chain (green sticks) and three on the light chain (cyan sticks). (**c**) Binding kinetics of SC57.32 Fab to human, cynomolgus monkey, rat and mouse MTf. K_D_: equilibrium dissociation constant calculated as k_d_/k_a_; k_a_ and k_d_: association and dissociation rate constants extracted from global fit of all sensorgrams, collected in triplicates and colored by analyte concentration (2.5, 5, 10, 20, 40 and 80 nM in red, green, blue, magenta, cyan, yellow, respectively). (**d**) Amino acid sequence alignment of human, cynomolgus monkey, rat and mouse MTf. Conserved epitope residues highlighted in green while divergent residues highlighted in red. N-glycosylation site indicated with arrow. Previously reported Alanine mutation sites that impaired SC57.32 binding indicated with asterisk. The variation of His 463 (that contacts Glu 50 of SC57.32 heavy chain) to Tyrosine in rat and mouse MTf prevents SC57.32 binding to rat and mouse MTf.

Our structure reveals that SC57.32 targets the C2 subdomain of the MTf C-lobe (Fig. 3A). In particular, ten residues of the Fab heavy chain and three of the light chain make direct contact with ten MTf residues (Arg 458, Arg 459, Asp 460, Ser 461, His 463, Arg 474, Val 564, Glu 565, Asn 566, Asp 598) as well as two GlcNAcs of the Asn 515 N-glycan (Fig. 3B and Supplementary Fig. S4). While N-glycans can exhibit variable heterogeneity, the SC57.32 Fab contacts monosaccharides that are part of the core N-glycan structure, which are present in every N-glycan^87^. SC57.32 Fab binding covers 1763.8 Å^2^ of total solvent accessible surface area on human MTf with the two GlcNAcs contributing 154.4 Å^2^, around 9% of the epitope surface area. Hydrogen bonds between the SC57.32 heavy chain and the two GlcNAcs contribute − 9.5 kcal/mol to the interaction, roughly 13% of the total energy according to Amoeba force field (Supplementary Fig. S4). Of note, the SC-005/SC57.32 epitope partially overlaps with the peptide used to transcytose IL-1RA across the blood–brain barrier^47^. This epitope is consistent with our previous report that Alanine mutation of two of these residues (Asp 460 and Asn 566) reduces SC57.32 binding and that Alanine mutation of His 463 completely abolishes it^65^. Out of the ten residues in direct contact, this is the only one not conserved in rat MTf and one of the two not conserved in mouse MTf (Fig. 3D). Consistent with the Alanine scan report, this single variation (His 463 to Tyr 463) is enough to prevent cross reactivity in rat and therefore also in mouse, where Asn 566 is also substituted by a Histidine. On the other hand, epitope conservation and cross-reactivity are fully retained in cynomolgus monkey MTf (Fig. 3C,D).

## Discussion

Melanotransferrin (MTf) is a surface-exposed and secreted protein of therapeutic interest in light of its use as a cancer target or as a drug-delivery system across the blood–brain barrier^41,60^. MTf is a bilobal transferrin-like glycoprotein capable of binding iron in the N- but not the C-lobe due to deviations in three iron coordination residues (Ser 421, Ala 478 and Ser 482). While there are examples of other bilobal transferrins that contain an active lobe and an inactive lobe^5,12^, our report constitutes the first transferrin structure of an active/inactive combination. We reveal a novel N–C lobe interface resulting in an inter-domain arrangement yet unseen in transferrins. While this conformation needs to be characterized further in solution, it is interesting to note that unlike most transferrins, MTf can be membrane bound via a GPI anchor^22,23^, raising the question on whether extending the C-lobe and its C-terminus away from the N-lobe and towards the plasma membrane can allow MTf to play unknown functions related to its lipid-bound state. In addition, it is worth noting that such N–C-lobe separation and larger C-lobe opening may coincide with the absence of inter-lobe regulation of iron uptake and release. While the interplay between N- and C-lobe in human serum transferrin has been reported to affect iron release rate of the C-lobe^11,88^, inactivity of the C-lobe might lead to a divergent conformation in MTf. More studies will be needed to assess whether MTf adopts other conformations in solution, as the one reported here may be one of several allowed. Finally, the characterization of the SC57.32 interaction with the glyco-epitope in the C2 subdomain of MTf offers visualization into the mechanism of action of SC-005 and provides a benchmark for future MTf-targeted applications.

## Supplementary information

Supplementary Figures.

## Data Availability

The Melanotransferrin/SC57.32 Fab structure has been deposited in the Protein Data Bank under the accession code: 6XR0. All other datasets generated during the current study are available from the corresponding author on reasonable request.
