# Supplementary material for: Complex of human Melanotransferrin and SC57.32 Fab fragment reveals novel interdomain arrangement with ferric N-lobe and open C-lobe
Source: Sci Rep. 2021 Jan 12;11:566. doi: 10.1038/s41598-020-79090-8 (PMC7804310; doi:10.1038/s41598-020-79090-8)
Supplement: Supplementary file 1 — Supplementary Figures. [file 41598_2020_79090_MOESM1_ESM.pdf]

Title: Complex of human Melanotransferrin and SC57.32 Fab fragment reveals novel interdomain arrangement with ferric N-lobe and open C-lobe

Kristyn Hayashi[1ⴕ], Kenton L. Longenecker[2ⴕ], Yi-Liang Liu[1], Bryan Faust[1], Aditi Prashar[1], Johannes Hampl[1], Vincent Stoll[2], Sandro Vivona[1*]

1 Research and Development, AbbVie Inc., South San Francisco, CA 94080
2 Research and Development, AbbVie Inc., North Chicago, IL 60064
ⴕThese authors contributed equally

*Corresponding author: Sandro Vivona
Email: sandrovivona@gmail.com
Phone: +1(650)305-1953
Address: 1505 O'Brien Drive, Menlo Park, CA 94025

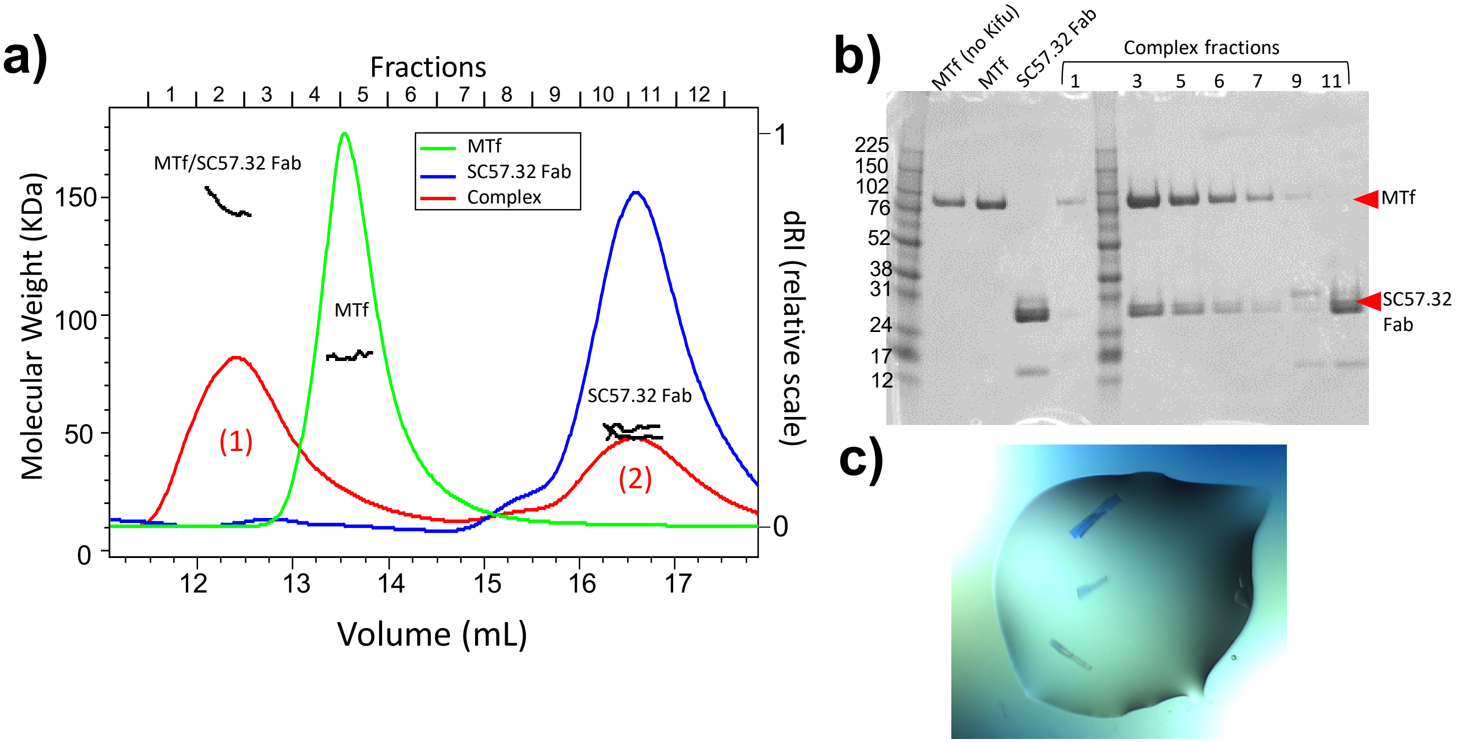

Supplementary Figure S1. Purification and crystallization of MTf/SC57.32 Fab complex. **a)** Size Exclusion Chromatography coupled with Multi-Angle Light Scattering (SEC-MALS) of free MTf (green), free SC57.32 Fab (blue) and MTf/SC57.32 Fab complex (red), of which peak 1 was used for the crystallization experiments. Molecular weight profiles are shown in black while differential refractive index (dRI) chromatograms are in colors. **b)** SDS-PAGE analysis of MTf and SC57.32 Fab isolated and complexed via SEC-MALS in a). A reference of MTf not treated with kifunensine is shown in second lane from left. Molecular markers are loaded in first and sixth lane from left to right. **c)** MTf and SC57.32 complex crystals in a 200 nL drop of 100 mM sodium acetate, pH 4.6 and 30% PEG300.

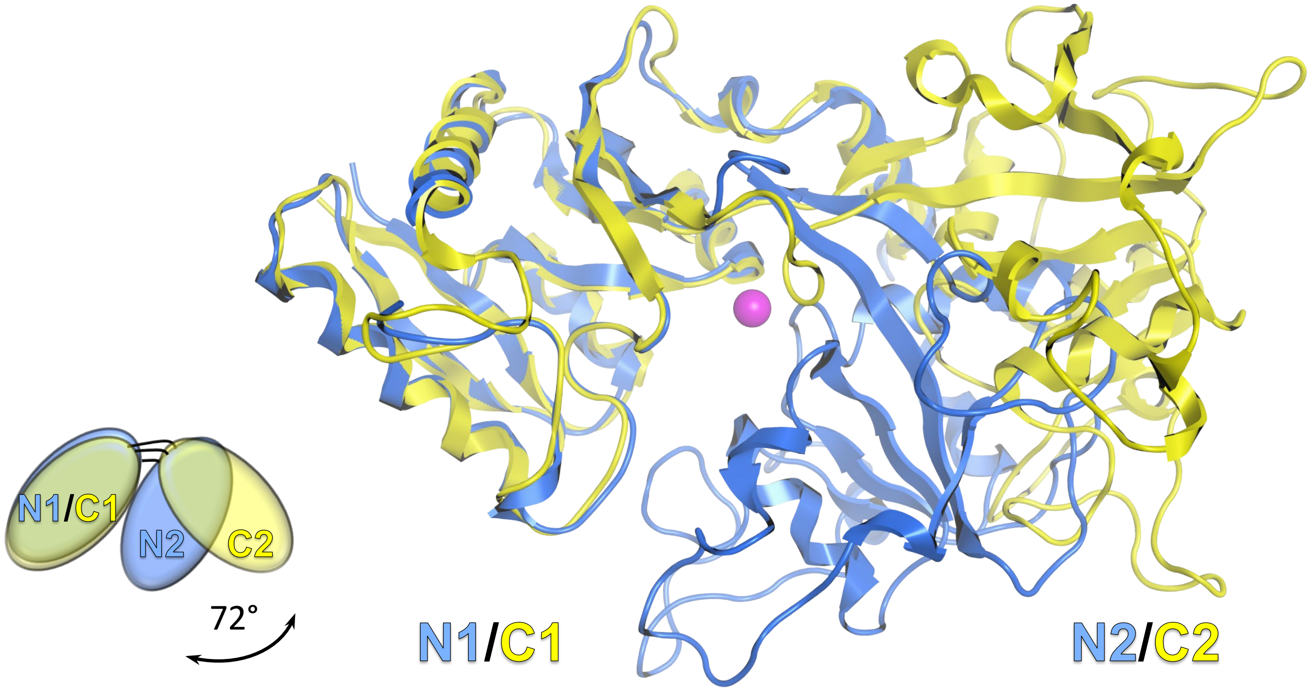

Supplementary Figure S2. Extent of MTf C-lobe opening in relation to iron-bound (magenta sphere) N-lobe. Superposition of open MTf C-lobe (yellow cartoon) on closed, ferric MTf N-lobe (blue cartoon) via subdomain 1 reveals a 72.1° swing of subdomain 2.

a)

Absorbance at 280 nm	Absorbance at 465 nm	MTf concentration (M)	Fe concentration (M)	Fe/MTf Molar Ratio
20.7	0.28	2.2E-04	1.3E-04	63.0%

b)

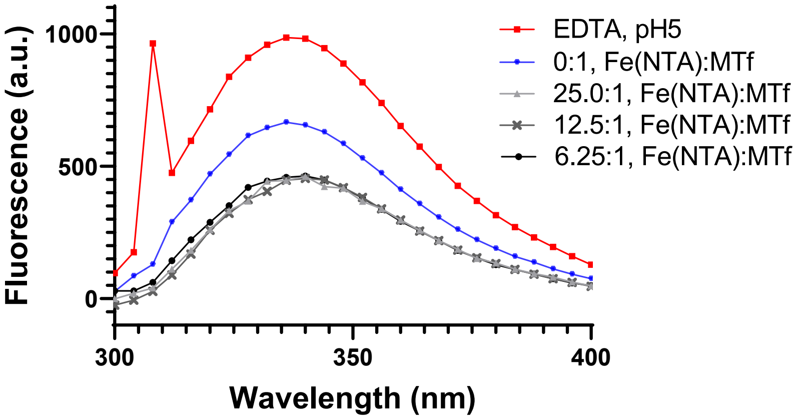

c)

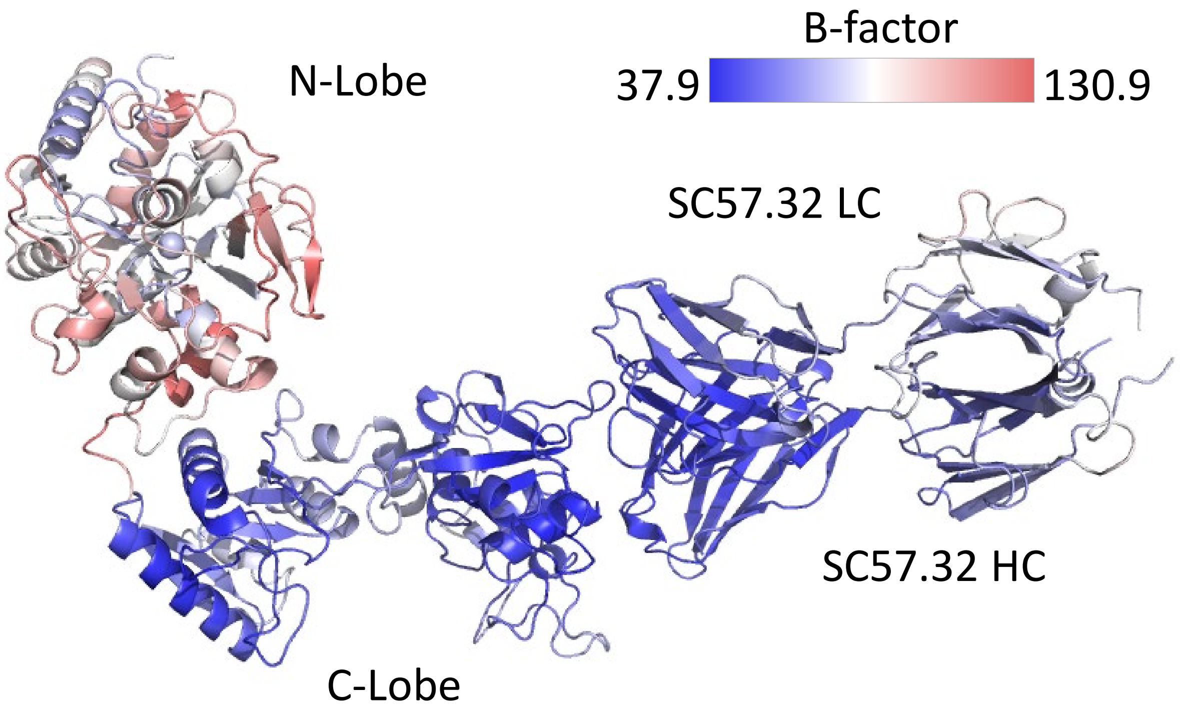

Supplementary Figure S3. a) Iron occupancy determination via UV-Vis spectroscopy. Absorbance of MTf at 280 and 465 nm provides molarity values of protein and iron (92970 and 2150 M^{-1} cm^{-1} were used as extinction coefficients, respectively) and indicate that 63% of the N-lobe of our MTf sample is loaded with iron. Values reported are average of six measurements.

b) Iron occupancy determination via fluorescence spectroscopy. 20 µg of MTf were incubated for one hour at room temperature with 1 mM EDTA, 100 mM sodium acetate pH 5, or with 100 mM ammonium bicarbonate, 50 mM HEPES pH 7.5 containing Fe(NTA) at 25, 12.5, 6.25 or 0 molar excess over MTf. All five samples were buffer exchanged into 100 mM ammonium bicarbonate, 50 mM HEPES pH 7.5 and their fluorescence spectra were recorded between 300 and 400 nm following excitation at 280 nm. Assuming 0% occupancy for the sample treated with EDTA (red) and 100% occupancy for the sample treated with 25 molar excess Fe(NTA) (grey), fluorescence at 340 nm indicates that our MTf sample (blue) is 63% saturated with iron. **c)** Cartoon representation of MTf in complex with SC57.32 Fab colored according to B-factor values, from 37.9 in blue to 130.9 in red. The figure was prepared in Pymol.

a)

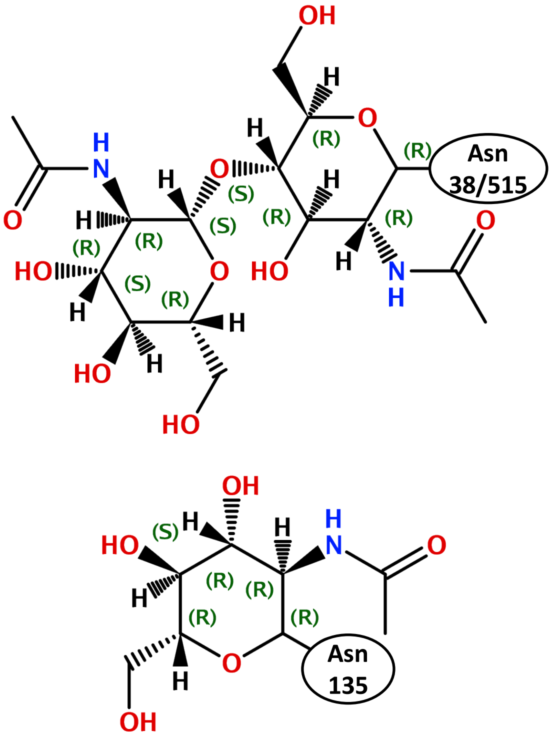

b)

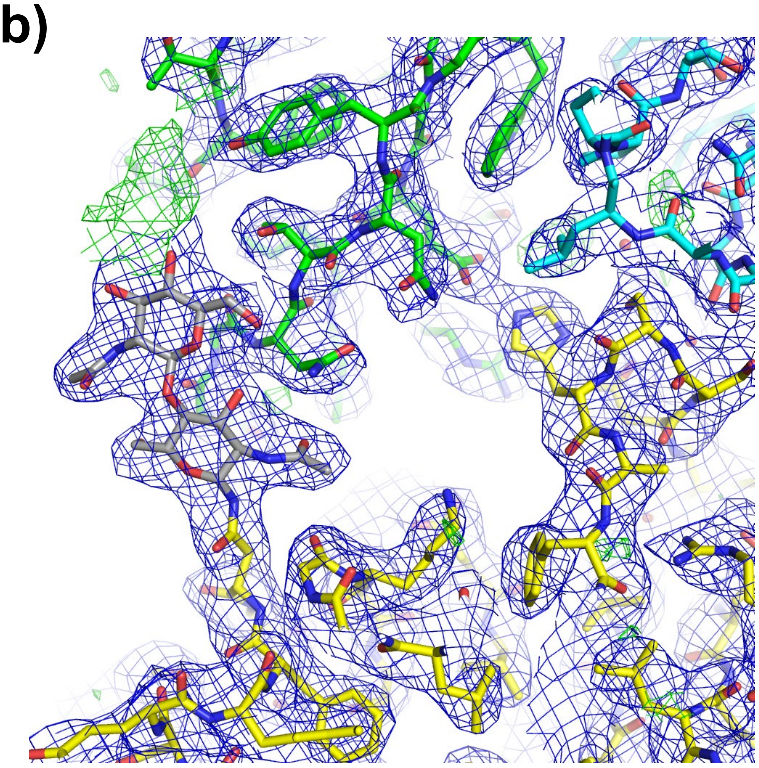

c)

Interaction Type	MTf	SC57.32 Fab	Distance (Å)	Energy (Kcal/Mol)
H bond	GlcNac #1	HC-N57	3.2	-3.2
H bond	GlcNac #1	HC-G55	3.2	-2.7
H bond	GlcNac #2	HC-N57	3.6	-0.7
H bond	GlcNac #2	HC-T58	3.4	-2.9
H bond	R458	HC-G101	3.3	-6.6
H bond	R459	HC-Y103	2.9	-4.4
Salt bridge	D460	HC-R33	3.7	-6.2
H bond	S461	LC-N92	2.6	-1.8
H bond	H463	HC-E50	2.9	-7.2
H bond	R474	HC-N31	3.3	-4.6
H bond	V564	HC-Y102	3.2	-0.7
H bond	N566	HC-D100	3.1	-1.4
Salt bridge	D598	LC-R53	3.2	-29.0
H bond	D598	HC-Y103	2.9	-4.2

Supplementary Figure S4. a) N-glycans structures of MTf. One N-acetylglucosamine (GlcNAc) is visible and covalently linked to Asn 135. Two GlcNAcs are depicted in the N-glycans attached to Asn 38 and Asn 515. **b)** Electron density map of SC57.32 glyco-epitope. SC57.32 heavy chain (green sticks) and light chain (cyan sticks) contact MTf (yellow sticks) and the N-glycan at Asn 515 (gray sticks). The 2Fo-Fc map is contoured at 1σ (blue) and an Fo–Fc map is contoured at 3σ (green). **c)** List of contacts between MTf and SC57.32 Fab. Cell shading colored according to b). Energy values obtained via Amoeba force field in MOE (Chemical Computing Group).
